# Double vitrification of embryos adversely affects clinical outcomes

**DOI:** 10.5935/1518-0557.20240014

**Published:** 2024

**Authors:** Chara Oraiopoulou, Mary Karagianni, Achilleas Papatheodorou, Olga Toumpa, Marianna Papadopoulou, Nicholaos Christophoridis, Panagiotis Drakopoulos, Alexia Chatziparasidou

**Affiliations:** 1 Embryology Department, Embryolab Fertility Clinic, Thessaloniki, Greece; 2 Embryolab Academy, Thessaloniki, Greece; 3 Clinical Department, Embryolab Fertility Clinic, Thessaloniki, Greece

**Keywords:** double vitrification, double cryopreservation, live birth rate, blastocyst

## Abstract

**Objective:**

To evaluate the impact of double embryo vitrification on clinical outcomes.

**Methods:**

This retrospective cohort study included data from January 2013 to March 2021. The study group included women aged 33.3±5.7 years with double-vitrified embryos (n=381), while the control group included women aged 32.1±6.7 years with embryos vitrified once (n=780), all transferred at the blastocyst stage. The primary endpoint was live birth rate (LBR), and secondary endpoints included percent positive βHCG test, clinical/ongoing pregnancy rates, miscarriage/biochemical pregnancy rates and birthweight.

**Results:**

LBR was significantly lower in double-vitrified embryos (30.2%) than in embryos vitrified once (45.6%, *p*<.05). Similarly, double-vitrified embryos were associated with significantly lower positive βHCG tests (46% vs. 63.3%, *p*<.05) and clinical (34.9% vs. 52.2%, *p*<.05) and ongoing pregnancy (31.3% *vs*. 47.3%, *p*<.05) rates compared to embryos vitrified once. However, biochemical pregnancy (double vitrified: 24.1% *vs*. vitrified once: 17.9%, *p*>.05) and miscarriage rates (double vitrified: 10.2% vs. vitrified once: 9.4%, *p*>.05), as well as mean birthweight (double-vitrified embryos: 2950g vs. embryos vitrified once: 2837g, *p*>.05) did not differ significantly between two groups. On a secondary comparison, amongst double-vitrified embryos, the subgroup that was cultured for more than 24 hours between warming and second vitrification achieved significantly higher positive βHCG tests (49%) and clinical pregnancy (38%) rates, compared to embryos re-vitrified on the same day of warming (31.8% and 20.5%, respectively, *p*<.05). Nevertheless, LBR did not differ significantly amongst these study-group embryos (embryos that remained in culture for more than 24 hours: 32.2% *vs*. embryos that were re-vitrified on warming day: 20.5%, *p*>.05).

**Conclusions:**

Double vitrification of embryos adversely affects clinical outcomes. However, it represents a valuable option concerning embryo wastage, with acceptable success rates.

## INTRODUCTION

Since vitrification was recognized as a high-efficiency method, it became a fundamental element in assisted reproductive technology (ART) (Edgar & Gook, 2012). The high survival rates obtained after vitrification in combination with the improved pregnancy rates of frozen embryo transfers (FET) (Roque *et al*., 2013) resulted in a trend towards elective freeze-all strategies (Blockeel *et al*., 2019), which often included vitrification of more than one embryo per vitrification device. Meanwhile, scientific societies (ESHRE & ASRM) and guidelines promoted single embryo transfer (SET) as the optimal practice to mitigate the risks of multiple pregnancies (The Practice Committee of the ASRM & the Practice Committee of the ASRT, 2013). As a result, a gradual shift of practice took place from double to single embryo transfers. However, there are still cases in which embryos are vitrified in groups of two or more per vitrification device. Thus, double vitrification may be decided as a means to salvage viable-looking embryos post ET that may contribute to a successful outcome in the future.

Although transfers of double-vitrified embryos are performed worldwide, it remains yet to be answered whether embryos, after double vitrification, exhibit the same reproductive performance as embryos vitrified once. Limited studies have been published to date, with differences in both cryopreservation methods and developmental stages in which cryopreservation was performed, with relatively conflicting results (Table 1). More specifically, there are studies including slow freezing as a first cryopreservation step and vitrification as a second one (Koch *et al*., 2011; Murakami *et al.*, 2011; Stanger *et al*., 2012; Lierman *et al*., 2014; Zheng *et al*., 2017; Hallamaa *et al*., 2021). Although pregnancy rates of double-cryopreserved embryos were lower than the rates of embryos cryopreserved once, only Zheng *et al*. (2017) concluded that live birth rate of double-cryopreserved embryos was significantly lower, with authors suggesting that double-cryopreserved embryos demonstrated comparable rates.

**Table 1:** Cryopreservation methods and embryo developmental stages included in published studies.

Author	Year published	Cryopreservation Method (1^s^t step)	Day of 1^st^ cryopreservation	Re-Cryopreservation Method (2^nd^ step)	Day of re- cryopreservation	Day of embryo transfers
Check *et al*.	2001	slow freezing	1 to 3	slow freezing	3	3
Kumasako *et al*.	2009	vitrification	1	vitrification	4 or 5	4 or 5
Murakami *et al*.	2011	slow freezing	1 to 3	vitrification	5	5
Koch *et al*.	2011	slow freezing	1 to 5	slow freezing / vitrification	3 to 5	_
Lierman *et al*.	2014	slow freezing	2 or 3	vitrification	6	5
Zheng *et al*.	2017	slow freezing	3	vitrification	5 to 7	5
Wang *et al*.	2021	vitrification	5 or 6	vitrification	5 or 6	5
Hallamaa *et al*.	2021	slow freezing / vitrification	2 or 3	vitrification	__	__
Li *et al*.	2021	vitrification	3 or 5	vitrification	5	5
Shen *et al*.	2023	vitrification	3	vitrification	5	5

There are four studies including vitrification in both cryopreservation steps (Kumasako *et al*., 2009; Wang *et al*., 2021; Li *et al*., 2021; Shen *et al*., 2023). In particular, Kumasako *et al*. (2009) demonstrated no significant difference in both clinical pregnancy and miscarriage rates, while Li *et al*. (2021) concluded that double-vitrification at the cleavage stage achieved comparable pregnancy outcomes to single vitrification, while procedures performed at the blastocyst stage led to reduced pregnancy rates. On the other hand, Wang *et al*. (2021) indicated an adverse effect of double vitrification on pregnancy, live birth and miscarriage rates, whereas Shen *et al.* (2023) found no effect of double vitrification in perinatal outcomes.

In our clinical practice, we perform double vitrification in certain cases: i) If two blastocysts were vitrified on the same vitrification device, but the patient decides to proceed with SET. ii) A previous standard of care stipulated the vitrification of all good-quality cleavage stage embryos on day three of their development. Nowadays, as we opt for blastocyst embryo transfers, we warm all available cleavage stage embryos, culture them to day 5 and then proceed with FET. iii) In unforeseen circumstances related to the patient’s condition, such as a positive COVID test post embryo warming. Thus, the option of double-vitrification offers the opportunity to increase cumulative pregnancy rates, while decreasing multiple gestation risk.

The aim of the present study was to evaluate the pregnancy and live birth rates of double-vitrified embryos compared to embryos vitrified once, and thus, to provide data that will help ART practitioners to guide patients facing this dilemma and expectations around the pregnancy outcomes after the transfer of a double-vitrified embryo. Moreover, the effect of embryo culture for at least one day between warming and second vitrification was compared to the effect of immediate re-vitrification on clinical outcomes.

## MATERIAL AND METHODS

### Study design

The assisted reproduction treatments included in this retrospective study were performed from January 2013 to March 2021 at Embryolab Fertility Clinic, Greece. The study group included double-vitrified embryos, while the control group included embryos from different patients that were vitrified once. In both study and control groups, all embryos were transferred at the blastocyst stage, at the same time period. Moreover, both PGT and cryopreserved oocyte cycles were excluded from the study. There was no variation in the cryopreservation method used, since all embryos were vitrified/warmed by the same vitrification/warming protocol.

Study design and data analysis were approved by the Scientific Council of Embryolab Fertility Clinic.

### Vitrification / warming procedure

All embryos were vitrified by the same vitrification/warming protocol, in an open vitrification system (Cryotec Reprolife, Tokyo, Japan). Briefly, embryos were exposed to equilibration solution (Es) containing 7.5% DMSO and 7.5% ethylene glycol for 15 minutes at room temperature (RT), and subsequently to vitrification solution (Vs) containing 15% DMSO, 15% ethylene glycol and 0.5 M sucrose, for 1 minute (Vit Kit-Freeze, Irvine Scientific, Fujifilm, Santa Ana, USA).

Warming was performed in a three-step procedure. The embryos were warmed in thawing solution (Ts) containing 1 M sucrose, for 1 minute, at 37^o^C, then placed in the dilution solution (Ds) containing 0.5 M sucrose, for 3 minutes at RT and in the washing solution (Ws - HEPES buffered medium), for 5 minutes at RT (Vit Kit-Thaw, Irvine Scientific, Fujifilm, Santa Ana, USA).

### Embryo culture

After warming, the embryos in the control group were placed into pre-equilibrated culture media (Sage 1-Step, Cooper Surgical, Ballerup, Denmark), at 37^o^C, 6% CO_2_, in a humidified atmosphere, for 2-4 hours until the time of transfer. After warming, the embryos in the study group were cultured for either 2-4 hours (blastocysts) or 1-3 days (cleavage-stage embryos to reach the blastocyst stage), until the time of re-vitrification. Similarly, double-vitrified embryos were kept in culture for 2-4 hours post second warming, until the time of transfer.

Blastocysts were evaluated before transfer, according to Gardner’s Grading System (Gardner & Schoolcraft, 1999). Embryos were grouped into two categories according to their grading: good quality blastocysts (grade AA, AB, BA) and low-quality blastocysts (grade BB, BC, CB). All transferred embryos in both study and control group were of expansion “3” or above. PGT was not performed in any transferred embryo, either in the control or in the study group.

### Endometrial preparation

In frozen embryo transfer cycles, the luteal phase was supported with estrogens (Cyclacur, Bayer, Weimar, Germany) starting from day 2-3 of the menstrual cycle and for at least ten days. Progesterone (Utrogestan, Besins Healthcare, Chatswood, Australia - Vasclor, Verifield, London, UK) was administrated when endometrial thickness was greater than 7mm, as described in the literature (Mackens *et al*., 2017).

### Clinical outcomes

Pregnancy was confirmed by assessing serum β-HCG levels: if the βHCG level was greater than 20 mIU/ml, the pregnancy test was considered positive. Each outcome measure was defined according to the Revised Glossary on ART Terminology (Zegers-Hochschild *et al*., 2009). More specifically, clinical pregnancy rate (CPR) was calculated as the number of pregnancies diagnosed based on presence of a heartbeat on ultrasound examination per total embryo transfer cycles, while ongoing pregnancy rate (OPR) was calculated as the number of pregnancies of more than 12 weeks expressed per total embryo transfer cycles. The main outcome measure was the live birth rate (LBR), defined as the number of deliveries that resulted in at least one live born baby per total embryo transfer cycles.

Biochemical pregnancy rate was calculated as the number of pregnancies diagnosed by positive βHCG test that did not develop to clinical pregnancy per total positive βHCG pregnancies. Finally, miscarriage rate was calculated as the number of clinical pregnancies that did not develop to ongoing pregnancies per total clinical pregnancies.

### Statistical analysis

The main objective of this study was to compare the study group’s proportion of binary outcome variable LBR to the control group’s LBR. Secondary outcomes were the binary variables of βhCG, CPR, OPR, miscarriage and biochemical pregnancy rate, as well as the count variable of birth weight. Mean age of female patients, mean number of transferred embryos per ET as well as number of good quality embryos per ET were detected as confounding variables between the two groups, through Poisson models. The Shapiro-Wilk test was used to check for normality, Poisson Regression models were used to perform the comparisons for confounders, and Logistic Regression models adjusted for the confounding variables were employed to investigate whether the proportions of the variables of interest differed between the two groups.

For the second part of the analysis, the study focused on the study group alone, where the patients were further divided into two subgroups depending on the presence or absence of culture before the embryos were cryopreserved for a second time. The same binary and count variables were of interest and only mean female patient age was considered as a possible confounding factor. Binary variables were compared using a chi-squared test, while for birth weight a Poisson Regression model was used.

The significance level was set to 0.05 and all tests were two-tailed. All analyses were done in SAS version 9.4.

## RESULTS

### Baseline characteristics

In the present study, 381 double-vitrified blastocysts were transferred in 252 patients in the study group, while 780 blastocysts vitrified once were transferred in 450 patients in the control group (Table 2). All blastocysts in both groups survived vitrification (100% survival rate). Eighty percent of the transferred re-vitrified blastocysts were of good quality, while in the control group 87% were rated as good quality blastocysts. The blastocysts that reached both grade AA/AB/BA and expansion 3 to 6, according to Gardner’s grading system, were described as “good-quality blastocysts”. The embryo transfers (ETs) resulted in 80 newborns in the study group and in 212 newborns in the control group.

**Table 2:** Baseline characteristics of study and control groups.

	Study Group (double vitrification)	Control Group (single vitrification)	*p*-value
**Patients (n)**	252	450	
**Female patient mean age** (at oocyte retrieval)	33.3 (±5.7)	32.1 (±6.7)	**0.007**
**Transferred blastocysts (n)**	381	780	
**Good quality blastocysts**	304 (80%)	680 (87%)	**0.001**
**Mean number of embryos per ET**	1.51 (±0.59)	1.73 (±0.51)	**0.028**
**Newborns**	80	212	

### Clinical results

In our population, double-vitrified embryos demonstrated significantly lower rates in positive βHCG tests (46% *vs*. 63.3% in control group, *p*<.05), clinical pregnancy (34.9% *vs*. 52.2% in control group, *p*<.05), ongoing pregnancy (31.3% *vs*. 47.3% in control group, *p*<.05) and live birth (30.2% *vs*. 45.6% in control group, *p*<.05) compared to embryos vitrified once (Table 3). However, biochemical pregnancy (24.1% in the study group *vs*. 17.9% in the control group, *p*>.05) and miscarriage rates (10.2% in the study group vs. 9.4% in the control group, *p*>.05) did not differ significantly, although there was a tendency for higher biochemical pregnancy rate in double-vitrified embryos. Furthermore, there was no significant difference in mean birthweight between the two groups (2950gr in the study group *vs*. 2837gr in the control group, *p*>.05).

**Table 3:** Demographic and clinical data.

	Study Group (double vitrification)	Control Group (single vitrification)	*p*-value
**Transferred blastocysts (n)**	381	780	
**Embryo transfers (n)**	252	450	
**Positive βHCG**	46% (116/252)	63.3% (285/450)	0.016
**Clinical pregnancy rate**	34.9% (88/252)	52.2% (235/450)	0.006
**Ongoing pregnancy rate**	31.3% (79/252)	47.3% (213/450)	0.012
**Live birth rate**	30.2% (76/252)	45.6% (205/450)	0.021
**Biochemical pregnancy rate**	24.1% (28/116)	17.9% (51/285)	0.248
**Miscarriage rate**	10.2% (9/88)	9.4% (22/235)	0.894
**Newborns (n)**	80	212	
**Mean birthweight (gr)**	2950	2837	0.459

In order to investigate the potential impact of culturing the embryos before a second vitrification, the study group was further analyzed and two subgroups were detected (Table 4):

**Table 4:** Clinical parameters and outcomes in study-subgroup A and study-subgroup B.

	Study Subgroup A (with culture)	Study Subgroup B (w/o culture)	*p*-value
**Transferred blastocysts (n)**	332	49	
**Embryo transfers (n)**	208	44	
**Positive βHCG**	49% (102/208)	31.8% (14/44)	**0.028**
**Clinical pregnancy rate**	38% (79/208)	20.5% (9/44)	**0.012**
**Ongoing pregnancy rate**	33.7% (70/208)	20.5% (9/44)	0.056
**Live birth rate**	32.2% (67/208)	20.5% (9/44)	0.088
**Biochemical pregnancy rate**	22.5% (23/102)	35.7% (5/14)	0.328
**Miscarriage rate**	11.4% (9/79)	0%	**0.001**
**Newborns (n)**	71	9	
**Mean birthweight (gr)**	2963	3222	0.211

i) subgroup A: 332 embryos (208 ETs) that were cultured for at least 24 hours between warming and second vitrification. The embryos were at the cleavage stage after the first warming, and were cultured to the blastocyst stage (24 to 72 hours); they were re-vitrified as blastocysts and warmed again for ET.

ii) subgroup B: 49 blastocysts (44 ETs) that were re-vitrified on the day of warming (up to 4 hours between warming and second vitrification) and warmed again for ET.

Embryos in study subgroup A performed significantly better than embryos in study subgroup B concerning both positive βHCG test (49% *vs*. 31.8%, respectively, *p*<.05) and clinical pregnancy rates (38% *vs*. 20.5%, respectively, *p*<.05). However, ongoing pregnancy (33.7% in subgroup A *vs*. 20.5% in subgroup B, *p*>.05) and live birth rates (32.2% in subgroup A *vs*. 20.5% in subgroup B, *p*>.05) did not differ significantly between the two subgroups. Furthermore, although biochemical pregnancy rate was higher in subgroup B, the difference was not significant between the two subgroups (22.5% in subgroup A *vs*. 35.7% in subgroup B, *p*<.05). On the other hand, the miscarriage rate was significantly higher in subgroup A (11.4% *vs*. 0% in subgroup B, *p*<.05), but the small sample size of subgroup B should be considered. Moreover, there was no difference in birthweight between the two subgroups (2963gr in subgroup A *vs*. 3222gr in subgroup B, *p*>.05). Furthermore, pregnancy rates (positive βHCG test, clinical pregnancy, ongoing pregnancy, live birth) of study subgroup A were compared to the corresponding control group’s rates, indicating that all rates in the control group were significantly higher than the ones in subgroup A (*p*<.05). Similarly, pregnancy rates in the control group were significantly different (*p*<.05) than the ones seen in study subgroup B.

## DISCUSSION

As the proportion of cryopreserved embryo transfer cycles has been growing over fresh treatments (European IVF-Monitoring Consortium *et al*., 2016), embryo cryopreservation has become an integral part of ART. Consequently, with the increasing number of patients opting for SET, supernumerary embryos that develop post warming/transfer are re-cryopreserved for future use. Although the practice of double cryopreservation has been performed worldwide, there are conflicting data concerning its impact on pregnancy rates. Our study sought to investigate the impact of double vitrification on pregnancy rates, live birth rates and perinatal outcome, compared to single vitrification. According to our data, CPR, OPR and LBR of double-vitrified embryos were significantly lower than the rates seen in single-vitrified embryos, while biochemical pregnancy rate, miscarriage rate and birthweight did not differ significantly. Moreover, amongst double-vitrified embryos, the subgroup that was cultured for at least 24 hours before second vitrification reached significantly higher positive βHCG test rates and CPR.

The present study includes the largest sample size reported to date and strengthens the evidence of lower positive βHCG rates (17.3% reduction, *p*<.05), CPR (17.3% reduction, *p*<.05) and OPR (16% reduction, *p*<.05) in double-vitrified embryo transfers, compared to transfers of single-vitrified embryos. This was also indicated in other studies (Wang *et al*., 2021; Li *et al*., 2021), in which vitrification was applied in both cryopreservation steps; Wang *et al*. (2021) reported a reduction of 28.2% (*p*<.05) in CPR, while Li *et al*. (2021) found an equally significant reduction. Additionally, Farhi *et al*. concluded that CPR was significantly reduced (28.2% reduction, *p*<.05) in re-cryopreserved embryo cycles, but the study applied slow freezing in both cryopreservation steps. Moreover, the only meta-analyses published to date concerning the effect of embryo re-cryopreservation on clinical outcomes concluded that double cryopreservation led to impaired IVF success rates (Wang *et al*., 2023). However, Kumasako *et al*. (2009), in a study using vitrification only, reported no adverse effect of double vitrification in CPR. Other studies suggest there is no significant impact on pregnancy rates from double cryopreserved embryos (Check *et al*., 2001; Koch *et al*., 2011; Murakami *et al*., 2011; Zheng *et al*., 2017; Hallamaa *et al*., 2021), nevertheless, Check *et al*. (2001) applied slow freezing only and cleavage stage embryo transfer, while Koch *et al*. (2011), Murakami *et al*. (2011), Zheng *et al*. (2017) and Hallamaa *et al.* (2021) included both slow freezing and vitrification in their studies. Thus, in general, the diversity of cryopreservation methods used and the differences in embryo developmental stages between the published studies makes the direct comparison of results debatable (Table 1).

Our primary endpoint measure was LBR, which was significantly reduced (15.4% reduction, *p*<.05) in re-vitrified blastocysts transfers, compared to transfers of blastocysts vitrified once. This is in line with the results of Zheng *et al*. (2017), which found a 10% reduction (*p*<.05) in LBR of double cryopreserved embryos (slow freezing followed by vitrification), despite the non-significant difference in CPR. Moreover, the studies of Wang *et al*. (2023) and Li *et al*. (2021) also found reduced LBR for double-vitrified embryos, while other published studies indicated no significant difference in LBR between doubleand single-cryopreserved embryos.

Biochemical pregnancy rate, miscarriage rate and birthweight were also analyzed in the present study, but no significant differences were found between singleand double-vitrified embryos. Apart from Zheng *et al*. (2017) and Wang *et al.* (2023), who found significantly higher rates of miscarriage in double cryopreserved embryos, the rest of the studies indicated no effect in either miscarriage rate or birthweight (Kumasako *et al*., 2009; Koch *et al*., 2011; Murakami *et al*., 2011; Hallamaa *et al*., 2021; Li *et al*., 2021; Shen *et al*., 2023).

Interestingly, when the study group was divided into two subgroups, we found that embryos re-vitrified on the day of warming (subgroup B) had significantly lower positive βHCG test (17.2% reduction, *p*<.05) and clinical pregnancy rates (17.5% reduction, *p*<.05), compared to embryos cultured for at least 24 hours before second vitrification (subgroup A). Moreover, although there was a tendency for lower LBR in subgroup B, the difference was not significant. The findings might be an indication that embryos in culture were allowed more time to recover from potential cryoinjury and thus performed better than embryos that were re-vitrified on the same day of warming.

According to our results, although double-vitrified blastocysts did not perform as well as blastocysts vitrified once, they survived the second warming and presented acceptable success rates. On the other hand, our findings showed that double vitrification had an adverse effect on clinical outcomes, as we observed a 15.4% reduction in LBR, which implies that although double-vitrified embryos retained their integrity, alterations on a molecular level or cell ultrastructure might cause decreased rates. Since embryo cryopreservation has been linked to cryodamage not visible on embryo morphology, as alterations in DNA integrity (Kopeika *et al*., 2015), in embryo transcriptome (Shaw *et al*., 2012), and in gene expression patterns affecting embryo developmental competence in bovine embryos (Gutierrez-Castillo *et al*., 2021), the decreased rates might reflect a cumulative effect of the passage of embryos through the cryopreservation procedure twice.

There are certain limitations in the present study. First, it was a retrospective study, since ethical issues would arise otherwise. Also, embryos were vitrified on different developmental stages at the first vitrification step. However, the same vitrification/warming protocol was used for all procedures, and all embryo transfers were performed on the blastocyst stage.

## CONCLUSION

In our population, double-vitrified embryos led to acceptable success rates and viable pregnancies. On the other hand, our findings showed an adverse effect of double vitrification in clinical outcomes. Thus, the present study supports re-vitrification as part of the management of supernumerary embryos post warming/transfer and provides a clear basis for the expected outcomes in terms of clinical pregnancy rates and LBR. Our findings may be used in discussions with patients to manage their expectations about ART.

In the context of refining procedures to achieve the highest possible rates, we would suggest implementing strategies to reduce the need for double vitrification, such as arranging the number of embryos per vitrification device according to the patient’s needs. Moreover, if double vitrification is required, offering embryos the maximum possible time in culture might be a valuable option. Further prospective studies are warranted to confirm these results.

## References

[r1] Blockeel C, Campbell A, Coticchio G, Esler J, Garcia-Velasco JA, Santulli P, Pinborg A. (2019). Should we still perform fresh embryo transfers in
ART?. Hum Reprod.

[r2] Check JH, Brittingham D, Swenson K, Wilson C, Lurie D. (2001). Transfer of refrozen twice-thawed embryos do not decrease the
implantation rate. Clin Exp Obstet Gynecol.

[r3] Edgar DH, Gook DA. (2012). A critical appraisal of cryopreservation (slow cooling versus
vitrification) of human oocytes and embryos. Hum Reprod Update.

[r4] Kupka MS, D’Hooghe T, Ferraretti AP, de Mouzon J, Erb K, Castilla JA, Calhaz-Jorge C, De Geyter Ch, Goossens V, European IVF-Monitoring Consortium (EIM), European Society of Human Reproduction and Embryology
(ESHRE) (2016). Assisted reproductive technology in Europe, 2011: results
generated from European registers by ESHRE. Hum Reprod.

[r5] Gardner DK, Schoolcraft WB. (1999). Culture and transfer of human blastocysts. Curr Opin Obstet Gynecol.

[r6] Gutierrez-Castillo E, Ming H, Foster B, Gatenby L, Mak CK, Pinto C, Bondioli K, Jiang Z. (2021). Effect of vitrification on global gene expression dynamics of
bovine elongating embryos. Reprod Fertil Dev.

[r7] Hallamaa M, Seikkula J, Willman S, Ollila H, Jokimaa V. (2021). Pregnancy potential and perinatal outcomes of embryos
cryopreserved twice: a case-control study. Reprod Biomed Online.

[r8] Koch J, Costello MF, Chapman MG, Kilani S. (2011). Twice-frozen embryos are no detriment to pregnancy success: A
retrospective comparative study. Fertil Steril.

[r9] Kopeika J, Thornhill A, Khalaf Y. (2015). The effect of cryopreservation on the genome of gametes and
embryos: principles of cryobiology and critical appraisal of the
evidence. Hum Reprod Update.

[r10] Kumasako Y, Otsu E, Utsunomiya T, Araki Y. (2009). The efficacy of the transfer of twice frozen-thawed embryos with
the vitrification method. Fertil Steril.

[r11] Li J, Xiong S, Zhao Y, Li C, Han W, Huang G. (2021). Effect of the Re-Vitrification of Embryos at Different Stages on
Embryonic Developmental Potential. Front Endocrinol (Lausanne).

[r12] Lierman S, Van den Abbeel E, De Sutter P. (2014). Vitrification of human blastocysts previously cryopreserved by
slow controlled-rate freezing at the cleavage stage. J Assist Reprod Genet.

[r13] Mackens S, Santos-Ribeiro S, van den Vijver A, Racca A, Van Landuyt L, Tournaye H, Blockeel C. (2017). Frozen embryo transfer: a review on the optimal endometrial
preparation and timing. Hum Reprod.

[r14] Murakami M, Egashira A, Murakami K, Araki Y, Kuramoto T. (2011). Perinatal outcome of twice-frozen-thawed embryo transfers: a
clinical follow-up study. Fertil Steril.

[r15] Roque M, Lattes K, Serra S, Solà I, Geber S, Carreras R, Checa MA. (2013). Fresh embryo transfer versus frozen embryo transfer in in vitro
fertilization cycles: a systematic review and meta-analysis. Fertil Steril.

[r16] Shaw L, Sneddon SF, Brison DR, Kimber SJ. (2012). Comparison of gene expression in fresh and frozen-thawed human
preimplantation embryos. Reproduction.

[r17] Shen X, Ding M, Yan Y, Huang C, Wang S, Zhou J, Xing J. (2023). Perinatal outcomes of singletons following double
vitrification-warming procedures: a retrospective study using propensity
score analysis. BMC Pregnancy Childbirth.

[r18] Stanger J, Wong J, Conceicao J, Yovich J. (2012). Vitrification of human embryos previously cryostored by either
slow freezing or vitrification results in high pregnancy
rates. Reprod Biomed Online.

[r19] The Practice Committee of the American Society for Reproductive
Medicine and the Practice Committee of the Society for Assisted
Reproductive Technology (2013). Criteria for number of embryos to transfer: a committee
opinion. Fertil Steril.

[r20] Wang M, Jiang J, Xi Q, Li D, Ren X, Li Z, Zhu L, Jin L. (2021). Repeated cryopreservation process impairs embryo implantation
potential but does not affect neonatal outcomes. Reprod Biomed Online.

[r21] Wang X, Mao R, Wang M, Long R, Jin L, Zhu L. (2023). The effect of recryopreservation on embryo viability and outcomes
of in vitro fertilization: a systematic review and
meta-analysis. Fertil Steril.

[r22] Zegers-Hochschild F, Adamson GD, de Mouzon J, Ishihara O, Mansour R, Nygren K, Sullivan E, van der Poel S, International Committee for Monitoring Assisted Reproductive
Technology, World Health Organization (2009). The International Committee for Monitoring Assisted Reproductive
Technology (ICMART) and the World Health Organization (WHO) Revised Glossary
on ART Terminology. Hum Reprod.

[r23] Zheng X, Chen Y, Yan J, Wu Y, Zhuang X, Lin S, Zhu J, Lian Y, Qiao J, Liu P. (2017). Effect of repeated cryopreservation on human embryo developmental
potential. Reprod Biomed Online.

